# Dysbiosis of urinary microbiota is positively correlated with Type 2 diabetes mellitus

**DOI:** 10.18632/oncotarget.14028

**Published:** 2016-12-19

**Authors:** Fengping Liu, Zongxin Ling, Yonghong Xiao, Longxian Lv, Qing Yang, Baohong Wang, Haifeng Lu, Li Zheng, Ping Jiang, Wei Wang, Lanjuan Li

**Affiliations:** ^1^ Department of Urology, The First Affiliated Hospital, School of Medicine, Zhejiang University, Hangzhou, Zhejiang, China; ^2^ State Key Laboratory for Diagnosis and Treatment of Infectious Diseases, The First Affiliated Hospital, School of Medicine, Zhejiang University, China; ^3^ Collaborative Innovation Center for Diagnosis and Treatment of Infectious Diseases, The First Affiliated Hospital, School of Medicine, Zhejiang University, China; ^4^ Yancheng Medical College, Yancheng, Jiangsu, China

**Keywords:** Akkermansia muciniphila, Escherichia coli, lactobacillus, type 2 diabetes mellitus, urinary microbiota, Immunology and Microbiology Section, Immune response, Immunity

## Abstract

Type 2 diabetes mellitus (T2DM) may be associated with altered urinary microbiota in female patients. We investigated alterations of urinary microbiota in Chinese female T2DM patients, and explored the associations between urinary microbiota and a patient’s fasting blood glucose (FBG), urine glucose (UGLU), age, menstrual status, and body mass index (BMI). Midstream urine was collected from 70 female T2DM patients and 70 healthy females. Microbial diversity and composition were analyzed using the Illumina MiSeq sequencing platform by targeting the hypervariable V3-V4 regions of the 16S rRNA gene. We found that bacterial diversity was decreased in T2DM patients. Increased Actinobacteria phylum was positively correlated with FBG, UGLU, and BMI; *Lactobacillus* abundance decreased with age and menopause; and increased *Lactobacillus* correlated positively with FBG and UGLU. Decreased *Akkermansia muciniphila* was associated with FBG and UGLU. *Escherichia coli* abundance did not differ between the two cohorts. Carbohydrate and amino acid metabolism was reduced in T2DM patients, which were associated with bacterial richness indices such as Chao1 and ACE. Detailed microbiota analysis of well-characterized T2DM patients and healthy controls indicate that Chinese T2DM female patients exhibit dysbiosis of urinary microbiota.

## INTRODUCTION

Type 2 diabetes mellitus (T2DM) accounts for 90% of diabetes [1]. T2DM is not due to insufficient use of insulin but due to insufficient insulin secretion and insufficient insulin action. Hospitalization rate for urinary tract infection (UTI) caused by diabetes is over twice as much as those caused by other factors [2]. Damage to the genitourinary system caused by diabetic neuropathy results in bladder dysfunction, and increases the probability of UTI [3]. High levels of urine glucose (UGLU) can favor a proper microenvironment for UTI due to increased bacterial overgrowth [4]. Female patients are known to have higher prevalence of UTI than males [5], which may be associated with the anatomical and structural differences in the urethra between genders.

General clinical practice dictates diagnosing UTI with a standard urine culture (SUC), then prescribing proper antibiotics based on the culture results. UTI is diagnosed when a known uropathogen surpasses 10^5^ colony-forming units (CFUs) per milliliter [6]. The premise of this diagnosis is based on the hypothesis that the bladder is sterile. SUC limitations include: 90% of bacteria cannot be cultured via standard culture media [7], SUC technique favors fast-growing bacteria but cannot consider the fastidious pathogens [8], and SUC techniques are unable to detect the presence of bacteria embedded within biofilms [9]. Fastidious pathogens may not lead to a UTI diagnosis because they are below the 10^5^ CFU/mL threshold, but can still cause urinary disorders [4].

Recent 16S rRNA sequencing results indicate that the urinary tract possesses bacteria, regardless of a patient's present urinary tract symptoms [10–23]. The main bacteria of females with asymptomatic bacteriuria are *Lactobacillus* [11, 12, 18, 22], *Gardnerella* [11, 13, 18], and *Prevotella* [11, 12]. Increased diversity of urinary microbiota in females undergoing stress urinary incontinence surgery was correlated with patients’ hormonal status and body mass index (BMI) [22]. Urinary microbiota in women with urgency urinary incontinence (UUI) was affected by UUI episodes, treatment response, and risk of post-treatment UTI [20]. Because microorganisms are present in healthy urinary systems, administering antibiotics to treat a UTI may be inappropriate. Antibiotics may kill the beneficial microbiota in the bladder, promote harmful bacteria to multiply, and result in dysbiosis.

With the occurrence of T2DM, the function of the urinary system can be impaired, as indicated by the urine composition, showing bladder dysfunction, increased UGLU, and renal urine net acid excretion [24–26]. We studied whether the difference in urinary microbiota between healthy controls (HCs) and T2DM patients can be detected, and if these differences are influenced by patients’ conditions, such as fasting blood glucose (FBG), UGLU, BMI, etc.

## RESULTS

### Cohort description

The women in both cohorts were similar in age, marital status, menstrual status, BMI, co-occurrence of coronary heart disease and hypertension, water intake, and asymptomatic bacteriuria (*p* > 0.05). T2DM cohort had higher FBG, UGLU, higher occurrence of hyperlipidemia, and more UTIs in the last year (*p* < 0.05) (Table 1).

**Table 1:** Descriptive data of participants

Parameter		Value for cohort (na)b or statistic		
		HCs	T2DM	*p*-value^c^
Age group (yrs) [no.(%)] ^d^				1.00
26-35		2 (2.86)	2 (2.86)	
36-45		6 (8.57)	6 (8.57)	
46-55		11 (15.71)	11 (15.71)	
56-65		17 (24.3)	17 (24.3)	
66-75		23 (32.9)	23 (32.9)	
76 and above		11 (15.7)	11 (15.7)	
Marital status [no. (%)]^d^				1.00
	Living with a spouse	64 (91.4)	64 (91.4)	
	Not living with a spouse	6 (8.6)	6 (8.6)	
Menstrual status [no. (%)]^d^				1.00
	Premenopausal	11 (15.7)	11 (15.7)	
	Postmenopausal	54 (77.1)	54 (77.1)	
	Hysterectomy	5 (7.1)	5 (7.1)	
Body mass index (kg/m^2^)		23.10 ± 4.49	23.87 ± 3.65	0.27
Co-occurrence of disease [no. (%)]				
	Coronary Heart Disease	8 (11.42)	13 (18.57)	0.34
	Hypertension	23 (32.86)	35 (50.00)	0.06
	Hyperlipidemia	7 (10.00)	18 (25.71)	0.03
Diabetes duration (yrs)		N/A	9.77 (± 7.49)	
FBG (mmol/L)		5.22 (± 0.61)	7.83 (± 2.35)	0.00
FBG > 10 mmol/L [no. (%)]		N/A	12 (17.1)	
UGLU POS [no. (%)] ^e^		0(0.00)	14 (20.00)	0.00
Taking metformin		N/A	70 (100.00)	
UTIs [no. (%)] ^f^		7 (10.00)	27 (38.57)	0.00
Water intake ^g^		2484.24 ± 99.20	2526.04 ± 108.02	0.78
Asymptomatic bacteriuria with E. coli POS [no. (%)]^e^		5 (7.14)	6 (8.57)	1.00

^a^ no. of subjects.

^b^ Mean ± SD or no.(%).

^c^ Pearson’s chi-square and Fisher’s exact tests were used with categorical variables. Independent *t*-test was used with continuous variables.

^d^ Variables used for individual matching.

^e^ POS indicates positive.

^f^ UTIs indicates episodes of UTI in the last year.

^g^ The amount of water intake included drinking water intake and dietary fluid intake, which was examined by the Chinese Food Frequency Questionnaire.

### Sequencing data

From 140 samples, a total of 8,602,818 high quality reads were produced, with a median read length of 441bp (range from 423 to 491). Those reads accounted for 78.91% of the valid reads (11,181,603 total sequences), with an average of 61,448 reads (range from 13,419 to 308,123) per barcoded sample for downstream analysis. The total number of unique sequences from the two cohorts was 2,501,986, and represented all phylotypes.

**Figure 1:**
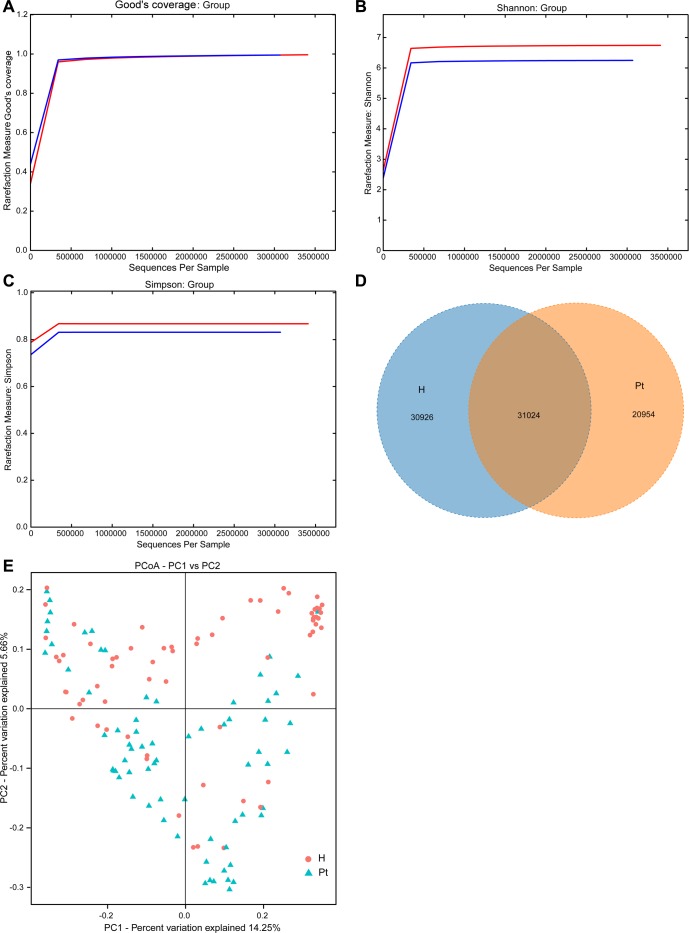
Structural comparison of urinary microbiota between two cohorts **A.** The Good’s coverage was used to assess sequencing depth. **B.** and **C.** The Shannon and Simpson Rarefaction curves were applied to estimate diversity. **D.** Venn diagram demonstrating overlap of OTUs in the urinary microbiota between the two cohorts. **E.** Principal coordinate analysis plot of the urinary microbiota based on the unweighted UniFrac metric. Red and blue lines and dots represent healthy controls and T2DM patients, respectively. H and Pt stand for healthy controls and T2DM patients, respectively.

Good's coverage indicated sufficient depth for the investigation of T2DM-associated urinary microbiota (Figure 1A). Both Shannon and Simpson indices illustrated the urinary microbiota diversity was lower in T2DM patients than in HCs (Table 2 and Table S1; Figure 1B and 1C). In the richness indices, ACE and Chao1 were lower in T2DM patients than those in HCs (Table 2 and Table S1). Interestingly, when correlation analyses between microbiota diversity and richness indices and participants’ age were conducted, no significant correlations were found (Table S2). A Venn diagram demonstrated 31,024 of the total 82,904 OTUs were shared between the two cohorts (Figure 1D). To measure the extent of the similarity of microbial communities, beta diversity was calculated using unweighted UniFrac and principal coordinate analysis (Figure 1E). The heatmap showed that the two cohorts were partially clustered (Figure S1).

**Table 2:** Comparison of richness and diversity estimation in HC and T2DM patients’ urine microbiota

Parameter^a^	HCs	T2DM	*p*-value
No. of reads	4,621,299	3,981,519	0.16
No. of OTUs ^b^	2692	1708	0.00
ACE ^c^	5446	3869	0.00
Chao1	2336	1500	0.00
Shannon	5.08	4.05	0.03
Simpson	0.72	0.67	0.23
Observed species	986	589	0.00
PD whole tree	97	64	0.00

^a^ parameters calculated by QIIME software;

^b^ the operational taxonomic units (OTUs) were defined at the 97% similarity level;

^c^ ACE indicates Abundance-based Coverage Estimator.

### Associations of urinary microbiota and T2DM

At the phylum level, the predominant sequences in the HCs were from Proteobacteria (58.01%), Firmicutes (22.41%), Bacteroidetes (9.33%), Actinobacteria (4.76%), and Acidobacteria (1.31%). The abundant sequences for the T2DM group belonged to Proteobacteria (51.63%), Firmicutes (24.31%), Bacterioidetes (13.07%), Actinobacteria (7.49%), and Thermi (0.80%). When the relative abundance of bacterial phylum was compared, Chloroflexi, Nitrospirae, and Gemmatimonadetes were more abundant in the HCs than those from the T2DM cohort (data not shown).

**Figure 2:**
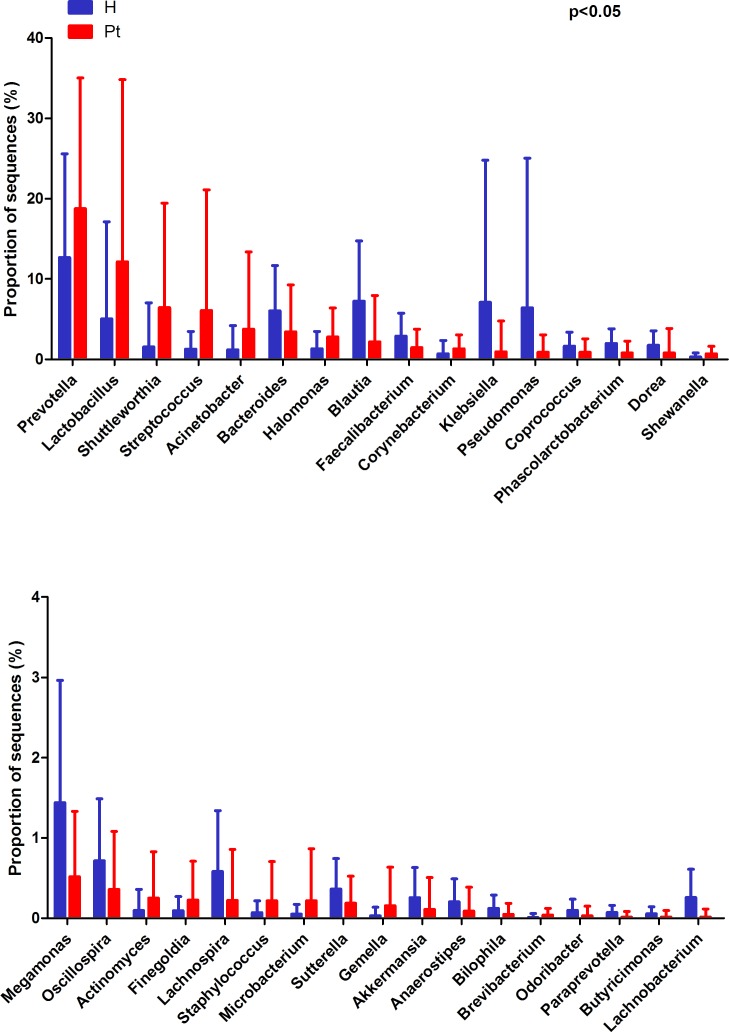
Genus-level OTUs different between the two cohorts (Mean ± SD) Welch’s *t*-test was used to compare the abundance at the bacterial genus level between HCs and T2DM patients. The different genera were assigned only to those presenting a minimum variation at a significant level [p (corrected) < 0.05)]. H and Pt represent healthy controls and T2DM patients, respectively.

At the genus level, HCs were mainly assigned to *Prevotella* (12.67%), *Blautia* (7.24%), and *Klebsiella* (7.07%). The most abundant genus was also *Prevotella* (18.76%) in T2DM patients, followed by *Lactobacillus* (12.15%) and *Shuttleworthia* (6.42%). Genera with different relative abundances between the two cohorts are listed in Figure 2, and their proportions are listed in Table S3 (*p* < 0.05). The relative abundance of *Gardnerella* was only 0.0018% and 0.0017% in the HCs and T2DM patients, respectively. Moreover, it only presented in 3 HCs and 5 T2DM patients. Only 18.57% (13/70) of the HCs and 17.14% (12/70) of the T2DM subjects presented *Escherichia* (*p* > 0.05).

The significantly different bacterial species are listed in Figure S2. The abundance of *Akkermansia muciniphila* was significantly higher in the HCs than T2DM subjects. Interestingly, *Escherichia* co*li* was not significantly more abundant in patients than HCs (0.008 ± 0.010 vs. 0.006 ± 0.009, p > 0.05).

### T2DM associated biomarkers

**Figure 3:**
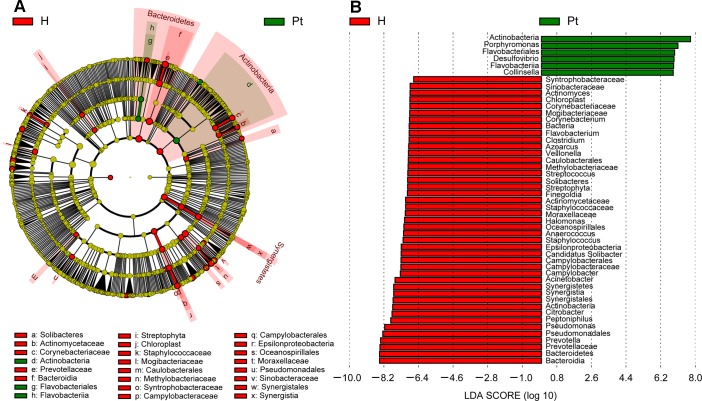
Cladogram showing differentially abundant taxa of microbiota **A.** LEfSe cladogram showed the most differentially abundant taxa between the two cohorts. Taxonomic cladogram obtained from LEfSe analysis of 16S sequences. Taxa enriched for HCs in red; T2DM enriched taxa in Green. The brightness of each dot is proportional to its effect size. **B.** Only taxa meeting an LDA threshold > 1.8 are shown. H and Pt represent healthy controls and T2DM patients, respectively.

To identify the specific bacteria taxa associated with T2DM, the urinary microbiota in the two cohorts were compared using LEfSe. A cladogram representative of the structure of the urinary microbiota and their predominant bacteria is shown in Figure 3A and 3B; the greatest differences in taxa between two cohorts are displayed. The data suggested that dysbiosis was extensive in T2DM patients. Actinobacteria, Flavobacteriales, and Flavobacteria could be used as potential distinguishing biomarkers.

### Urinary microbiota affected by participants’ conditions

The relative abundance of Actinobacteria and *Lactobacillus* increased with FBG and UGLU in the T2DM cohort (Figure S3A, S3B, S4A, S4B). In contrast, *A. muciniphila* was lower in the T2DM patients with FBG > 10 mmol/L and UGLU positive results (Figure S5A and S5B).

**Figure 4:**
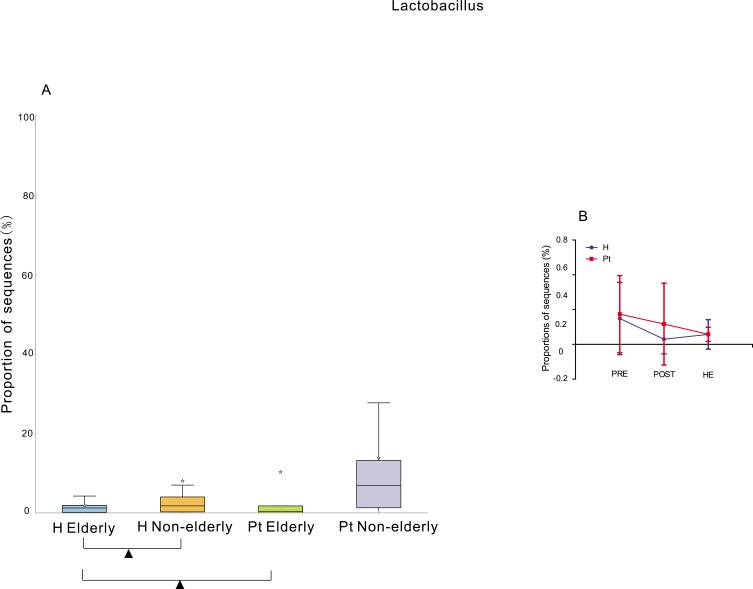
Relative abundance of Lactobacillus associated with age and menstrual status **A.** Box plot showing the distribution in the proportion of *Lactobacillus* assigned to samples from H elderly, H non-elderly, Pt elderly, and Pt non-elderly. Triangles represent a significant difference was found between H elderly and H non-elderly, and between H elderly and Pt elderly. The median value is shown as a line within the box, and the mean value as a star. **B.** The difference among pre-menopausal status (PRE), hysterectomy (HE), and post-menopausal (POST) status groups. ANOVA test was applied, and Benjamini-Hochberg FDR was used as a correction approach to control the false discovery rate, p (corrected) < 0.05 was considered significant. H and Pt mean healthy controls and T2DM patients, respectively.

The elderly (> = 65 years old) HCs had significantly lower abundance of *Lactobacillus* than the non-elderly (< 65 years old) HCs [38], but the elderly T2DM patients did not show significantly decreased *Lactobacillus* abundance compared to the non-elderly T2DM patients. The healthy elderly had significantly lower abundance of *Lactobacillus* than the elderly T2DM patients. In contrast, the healthy non-elderly subjects did not show a significantly lower abundance than the non-elderly T2DM patients (Figure 4A). The HC subgroup with the lowest *Lactobacillus* abundance was the post-menopause subgroup, and the lowest one from the T2DM cohort was the hysterectomy subgroup (Figure 4B). Moreover, the abundance of *Lactobacillus* in the HCs was negatively correlated to the years after menopause (r = -0.33, *p* < 0.01). No correlation was found in the T2DM patients (r = -0.06, *p* > 0.05).

*A. muciniphila* was most abundant in the obese HC group, and least abundant in the obese T2DM group (Figure S6A). Actinobacteria was not only enriched in T2DM patients, but also increased with their BMI (Figure S6B).

No difference was noted between the asymptomatic bacteriuria samples with *E. coli*, and the non-asymptomatic bacteriuria samples (0.02 ± 0.01, 0.01 ± 0.01, *p* > 0.05). In addition, the abundance of the *E. coli* was correlated with water intake (r = 0.26, *p* < 0.01). No correlation with UTIs was found (r = 0.11, *p* > 0.05).

### Urinary microbiota associated with metabolism

**Figure 5:**
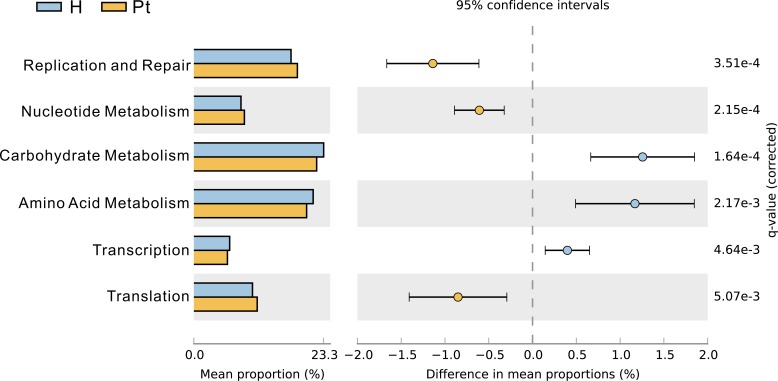
Clusters of orthologous group categories Clusters of orthologous group categories reveal metabolic functions that were enriched in urinary microbiota from the two cohorts. P values were based on White's nonparameteric *t*-test with the Benjamini-Hochberg FDR false discovery rate correction approach.

We observed that HCs and T2DM patients had differing patterns of enrichment in terms of clusters of orthologous group categories. Carbohydrate and amino acid metabolism was damaged in T2DM patients (Figure 5), and they were positively correlated with Chao1 and ACE (r = 0.53, *p* < 0.01: r = 0.56, *p* < 0.01; r = 0.54, *p* < 0.01: r = 0.58, *p* < 0.01).

## DISCUSSION

In the present study, a reduction of urinary microbial diversity and overall richness in T2DM patients was detected in contrast to HCs (Figure 1B, 1C, and Table 2). The alteration was similar to previous studies that compared the urinary microbiota of HCs to UUI and interstial cystitis patients [13, 18], which also indicated that non-healthy patients had reduced diversity and richness. Additionally, it was similar to another study in which the urinary microbiota richness was lower in UTI patients than non-UTI subjects [21]. However, it was dissimilar to a recent study, which revealed that UUI patients had slightly higher diversity than controls [28]. The diversity was not correlated with age (Table S2), and was inconsistent with a previous study in which women over 70 had 75% fewer bacteria than women aged 20-49 [15]. The Venn diagram demonstrated less bacterial phylotypes in T2DM patients compared to HCs (Figure 1D). It also suggested the occurrence of T2DM has altered urinary microbiota composition. The principal coordinate analysis and heatmap showed that most of the samples from the HCs and T2DM patients can be clustered together (Figure 1E and Figure S1), although not completely clustered. The inter-individual variation suggests that other factors may affect urinary microbiota besides T2DM. This phenomenon of inter-individual variation is also demonstrated by recent studies on urinary microbiota [10, 12, 16, 17, 28].

The most abundant phylum in both cohorts was Proteobacteria, as opposed to Firmicutes, which has commonly been found to be the most abundant bacteria [11, 13, 18, 22, 28]. The relative abundance of Proteobacteria was higher in the HCs than the T2DM cohort. This result is in contrast to similar studies which found the HCs to have lower relative Proteobacteria abundance when compared to non-healthy controls [21].

In the present study, the most abundant bacteria for both cohorts were *Prevotella*, while *Lactobacillus* was the second most abundant bacteria. In previous studies, *Lactobacillus* was the most abundant genus, while *Prevotella* was the second and/or third most abundant, or even less [11, 18, 20]. This shift may be associated with ethnicity, since there is a difference between Caucasian and Chinese gut microbiota [29]. *Prevotella* was enriched in T2DM patients (Figure 2), and similar studies have shown *Prevotella* is higher in non-healthy patients when compared to controls [13, 18]. *Prevotella* has been considered pathogenic in the vaginal microbiome [30], and future studies should explore whether *Prevotella* also acts pathogenic in the infections in diabetes [31].

*Lactobacillus* was increased in T2DM patients (Figure 2), and abundance increased with patients’ FBG and UGLU levels (Figure S4A, S4B). *Lactobacillus* is probiotic, and can produce organic acid and hydrogen peroxide that create an inhospitable environment for pathogenic bacteria. Treatment with *Lactobacillus* can reduce the incidence of UTIs [32], and can reduce FBG, homocysteine, and interleukin-6 which play roles in preventing infection for diabetic patients [33–35]. In addition, the higher proportion of *Lactobacillus* can increase insulin secretion [36]. Therefore, increased *Lactobacillus* might be considered a protective effect on T2DM patients from infections and reducing blood glucose.

The elderly and postmenopausal participants from both the HC and T2DM cohorts had a lower abundance of *Lactobacillus* (Figure 4A, 4B). This is similar to Karstens et al. study, in which *Lactobacillus* dominated samples from premenopausal female [28], and higher frequency of *Lactobacillus* was detected in samples from premenopausal female and postmenopausal female on exogenous hormone therapy [22]. The level of free glycogen in post-menopausal women is lower than that in pre-menopausal women, caused by the reduction of estrogen [37], and levels of free glycogen are associated with the levels of *Lactobacillus* in the vagina [37, 38]. The abundance of *Lactobacillus* in urine can be affected by the levels of vaginal *Lactobacillus*, because the urinary meatus is near the vagina. Thus, the reduction of *Lactobacillus* might be caused by the decline of estrogen with the arrival of menopause [39]. However, *Lactobacillus* did not decrease with the number of years after menopause in the T2DM cohort. These trends might suggest that T2DM inhibited the reduction of *Lactobacillus*, or reducing estrogen levels in elderly diabetes patients only plays a limited role in regulating the reproduction of *Lactobacillus*. Future study focusing on whether *Lactobacillus* in T2DM patient responds differently from HCs or estrogen regulating the reproduction of *Lactobacillus* in T2DM is different from HCs is needed.

The T2DM hysterectomy subgroup had the lowest *Lactobacillus* among pre-menopause, hysterectomy, and post-menopause patients (Figure 4B), while this was not the case in controls. This might be due to vaginal microbiota dysbiosis caused by hysterectomy, including declined *Lactobacillus* [40]. Adjacent to the urinary tract is the reproductive system, and it is possible that *Lactobacillus* of urine migrates from the vagina.

A decrease of *Akkermansia* and *A. muciniphila* in T2DM patients was found (Figure 2 and Figure S2), and the abundance of *A. muciniphila* decreased with patients’ FBG and UGLU (Figure S5A, S5B). Intestinal studies have also shown that *A. muciniphila* was involved in glucose homeostasis [41], but its abundance was not correlated with BMI (Figure S6A). This suggests that *A. muciniphila* cannot be considered as “lean” bacteria in urine.

Actinobacteria, a representative member of phyla in healthy female urine [15], was higher in T2DM patients than HCs. Moreover, it has been identified as a biomarker distinguishing T2DM and HCs (Figure 3A, 3B), and has a higher gut prevalence in obese patients when compared to lean subjects [42]. Our study also demonstrated that Actinobacteria increased in patients with a higher BMI (Figure S6B). Actinobacteria increased with patients’ FBG and UGLU (Figure S3A, S3B), so it could be used as a predictor of diabetic progress.

Since *E. coli* can adhere better in diabetic females than in healthy subjects [43], the abundance of *E. coli* in T2DM patients’ urine should be higher than controls. However, no difference was found in the abundance of *E. coli* between the two cohorts. Additionally, no difference was detected between *E. coli* positive and negative samples in T2DM patients. It is possible that *Lactobacillus*, or other probiotic bacteria, in urine could inhibit the growth of *E. coli* [44]. The high abundance of *Lactobacillus* in patients might impair the ability of *E. coli* to adhere to uroepithelial cells.

*Gardnerella*, a genus representing one of the major urotypes in previous studies [11,18,20], had decreased abundance and prevalence in both cohorts, despite being a major component of vaginal microbiota [45]. The low abundance and prevalence might be related to the MMSU technique designed in our study. Because a genuine midstream urine was obtained, the risk of *Gardnerella* contaminating the specimen was low. The abundance of *Escherichia* was less than a previous study that collected urine with transurethral catheter or suprapubic aspiration [14]. This result might suggest that the MMSU can avoid bacterial contamination.

Carbohydrate and amino acid metabolism was damaged in T2DM patients (Figure 5), and correlated with patients’ bacterial diversity. This suggests that if carbohydrate and amino acid metabolism is improved in patients, urine microbiota composition will be enhanced. Moreover, therapy based on urinary microbiota can be used to evaluate and modulate a patient's metabolism.

Considering urine samples are at risk of contamination by bacteria of the female vagina and gut [14], we modified the aseptic technique used in the transurethral catheter. In addition, we designed a four-tube collection method which guaranteed that true midstream urine could be obtained. A previous study described that the rate of urine bacterial DNA detected for sequencing was about 33%-86% [16, 20, 46]. We applied magnetic-based beads DNA extraction method with adding lysis buffer [23] and only 1.43% (2/140) failed to provide sufficient DNA for sequence.

Our study did contain some limitations, such as including only female T2DM patients, so the influences of sex hormones on urinary microbiota could not be ruled out. Participants could not recall whether they took antibiotics over the last 6 months, and we could not collect the information in their medical records. We cannot judge long term effects of antibiotics on urinary microbiota [47]. Lastly, all the T2DM participants were treated with metformin, so we were unable to compare the effects of insulin and metformin treatment on the urinary microbiota (since the intestinal microbiota is affected by metformin) [48]. A useful future study would involve exploring the urinary microbiota of participants taking these two medications.

## CONCLUSIONS

We demonstrated that microbiota dysbiosis may be associated with T2DM. Secondly, the relative abundance of some key bacteria in T2DM patients was different than in the HCs, and the relative abundancies were affected by the patients’ characteristics. Lastly, there was an interdependency between urine microbiota and the patients’ metabolism. Future studies should focus on how the urinary microbiota affects patient's characteristics such as FBG and UGLU.

## MATERIALS AND METHODS

### Study design

The matched case-control study enrolled 70 patients with T2DM patients and 70 HCs from June 2015 to January 2016 from the Department of Endocrinology, the First Affiliated Hospital, School of Medicine, Zhejiang University (Table 1). The diagnostic criteria for T2DM are based on recommendations from the World Health Organization: fasting blood glucose (FBG) ≥ 7.0 mmol/L, or 2-h plasma glucose ≥ 11.0 mmol/L [49]. HCs were from local communities, had never been diagnosed with diabetes, and had a FBG ≤ 6.1 mmol/L [49]. The HCs were matched with the T2DM patients for age, marital status, and menstrual status (Table 1). A senior nurse assessed weather the participants met the inclusion criteria for the T2DM and HC cohorts. Written informed consent was obtained from participants prior to enrollment, with the approval of the Ethics Committee of the First Affiliated Hospital, School of Medicine, Zhejiang University (Reference Number: 295). The following criteria from participants’ medical records and/or complaints were used to exclude subjects: UTI in the previous month; use of antibiotics, probiotics, prebiotics, or synbiotics in the previous 3 months; unable to complete the questionnaire; menstruation; urinary incontinence; known anatomic urinary tract abnormalities (e.g. cystoceles, hydronephrosis, renal atrophy, or neurogenic bladder); urinary catheter.

### Sample collection and processing

Before urine sampling, the participants were instructed to use the modified mid-stream urine (MMSU) collection technique which was composed of disinfection techniques and four-tube collection methods (Supplemental Protocol 1). The first urine of the day was collected, immediately placed on ice, transferred to the laboratory within 15 minutes, and stored at -80°C [50]. Urine from Tube 2 and Tube 3 were used for urinalysis and SUC. Based on the guidelines established by the China Ministry of Health, urine culture was the reference method to determine specimen contamination [51].

Asymptomatic bacteriuria is defined as the presence of two consecutive MMSU specimens with isolations of the same bacterial strain at > 10^5^ CFU/mL [52]. If asymptomatic bacteriuria was confirmed, the second urine sample was used for bacterial sequencing. Additionally, FBG was measured on the same day as urine sample collection. After the participants were recruited into the HC or T2DM cohort, T2DM patients were divided into two subgroups: FBG ≤ 10 mmol/L group (well controlled group) and FBG > 10 mmol/L group (poor controlled group) [53]. A self-report questionnaire was used to collect demographic characteristics, and the Chinese Food Frequency Questionnaire was used to examine water intake [54].

### DNA extraction, PCR, and MiSeq sequencing

Total DNA was extracted from the pellet of urine from Tubes 2 and 3, and 40 mL of urine was aspirated from each tube, separated into three sections, and injected into three 15 mL sterile centrifuge tubes. Each tube was pelleted by centrifugation at 4,000 × g for 15 min at 4°C. 10 mL of the supernatant was decanted, and the pellet was obtained by centrifugation for 15 min at 4,000 × g at 4°C. The pellet was transferred into a 2 mL sterile centrifugation tube which contained 500 μL of lysis buffer [23]. The tube was kept at -80°C until DNA extraction. Magnetic bead isolation of genomic DNA from bacteria was performed per the manufacturer's protocol with minor modifications (Supplemental Protocol 2) [23]. The concentration of extracted DNA was determined by using a Nanodrop ND-1000 spectrophotometer (Thermo Electron Corporation, USA); its integrity and size were checked by 1.0 % agarose gel electrophoresis containing 0.5 mg/mL ethidium bromide. The DNA complex was placed at -20°C until PCR amplification. Two of the 140 samples failed to provide sufficient DNA for sequencing, so we recruited two subjects who had the same attributes as the former ones, and obtained sufficient DNA for sequencing. The 16S rRNA gene V3-V4 regions were PCR-amplified from microbial genome DNA (forward primer, 5′-ACTCCTACGGGAGGCAGCAG-3′; reverse primer, 5′-GGACTACHVGGGTWTCTAAT-3′) [55]. Negative DNA extraction controls (lysis buffer and kit reagents only) were amplified and sequenced as contamination controls. The amplicons were normalized, pooled, and sequenced on the Illumina MiSeq platform using a V3 reagent kit with 2×300 cycles.

### Bioinformatic and statistical analysis

Sequencing reads were processed using QIIME (version 1.9.0), and included additional quality trimming, demultiplexing, and taxonomic assignments. Profiling of predictive urine microbiota was analyzed by using PiCRUSt based on 13 August 2013 Greengenes database [56]. KW rank sum test and pairwise Wilcoxon test were used for the identification of the different markers, and LDA was used to score each feature in the LEfSe analysis. Index of alpha diversity was calculated with QIIME based on sequence similarity at 97%. Beta diversity was measured by unweighted UniFrac distance, which was also calculated by QIIME. Hierarchical clustering was performed, and a heatmap was generated using a Spearman's rank correlation coefficient as a distance measure and a customized script developed in the R statistical package. The output file was further analyzed using Statistical Analysis of Metagenomic Profiles software package (version 2.1.3) [57].

To obtain insight into the possible functional pathways that differ between T2DM and HCs, we used PiCRUSt to calculate contributions of various OTUs to known biological pathways using Kyoto Encyclopedia of Genes and Genomes (KEGG) databases [56]. The pathways that were nonprokaryotic, had fewer than 2 sequences in each cohort, or had a difference in mean proportions less than 0.1% was excluded from analysis [23].

Statistical analysis was performed using the SPSS data analysis program (version 21.0) and Statistical Analysis of Metagenomic Profiles software. For continuous variables, independent *t*-test, Welch's *t*- test, White's nonparametric *t*-test, and Mann-Whitney *U*-test were applied. For categorical variables between groups, using either the Pearson chi-square or Fisher's exact test, depending on assumption validity. For taxon among subgroups, ANOVA test was applied (Tukey-Kramer was used in Post-hoc test, Effect size was Eta-squared) with Benjamini-Hochberg FDP false discovery rate correction [58, 59]. All tests of significance were two-sided, and *p* < 0.05, or corrected *p* < 0.05, was considered statistically significant.

### Accession number

The sequence data from this study are deposited in the GenBank Sequence Read Archive with accession number SRP 087709.

## SUPPLEMENTARY MATERIAL FIGURES AND TABLES








