# Supplementary material for: Dysbiosis of urinary microbiota is positively correlated with Type 2 diabetes mellitus
Source: Oncotarget. 2016 Dec 19;8(3):3798–810. doi: 10.18632/oncotarget.14028 (PMC5354796; doi:10.18632/oncotarget.14028)
Supplement: Supplementary file 1 [file oncotarget-08-3798-s001.pdf]

Dysbiosis of urinary microbiota is positively correlated with Type 2 diabetes mellitus

Supplementary Material

Protocol 1: Midstream urine collection technique

(a) Opened Pvp-I Antiseptic (Dian'erkang, Shanghai, China) and poured onto the sterile cotton balls which were placed in a 40 mL sterile sputum cup; (b) opened the four 50 mL sterile centrifuge tubes which were labeled with numbers 1, 2, 3, and 4, respectively (Tube 1, Tube 2, Tube 3, and Tube 4), and placed the interior of the cover upwards, did not allow participants to touch the interior and the edge of the tube or the cover during this procedure; (c) pulled pants down to the knees and squat over a wash bowl or toilet pan, with spread legs; (d) disinfected thumb, middle, and index finger of both hands with Pvp-I Antiseptic twice; (e) used the dominant hand to pick up a cotton ball, cleaned the far labial fold, starting from above the meatus down towards the rectum. Discarded cotton ball without crossing the sterile field; repeated cleaning near labial fold and down center of meatus. Hold labia apart to prevent labia minora from falling back over meatus. After disinfection, participant initiated voiding the urine into Tube 1, until it reaches one-half tube. Then, without stopping the flow of urine, void urine into Tube 2, Tube 3 and Tube 4 in order. The remaining was voided to the wash bowl or toilet. The only difference between Tube 2 and the others was that this tube should be filled at the labeled line which represents 40 mL of urine. After training, we asked the participant to demonstrate the MMSU technique to assess their competency to perform the procedure.

Protocol 2: DNA isolation

The tube was placed in liquid nitrogen for 1 min, and transferred into a water bath at 65°C for 5 min, with vigorous mixing. This last process was repeated three times with a final bath for 30 min. 50 μL *Agencourt AMPure XP* (Beckman Coulter, USA) was added to 100 μL of the urine pellet, vortexed for 30 sec, and incubated for 5 min at room temperature. The tube was placed into a magnetic separator for 5 min, and DNA was bound to magnetic beads which were drawn to the wall of the microcentrifuge tube. The supernatant was carefully removed without disrupting the magnetic beads. The sample was washed twice with 200 μL 80% ethanol for 30 sec, being placed on a magnet separator between each washing. The purified DNA was eluted with 50 μL ddH_2O for 1 min. The beads, now released from the DNA, were collected with the magnet. The DNA-containing supernatant was transferred to a clean tube.

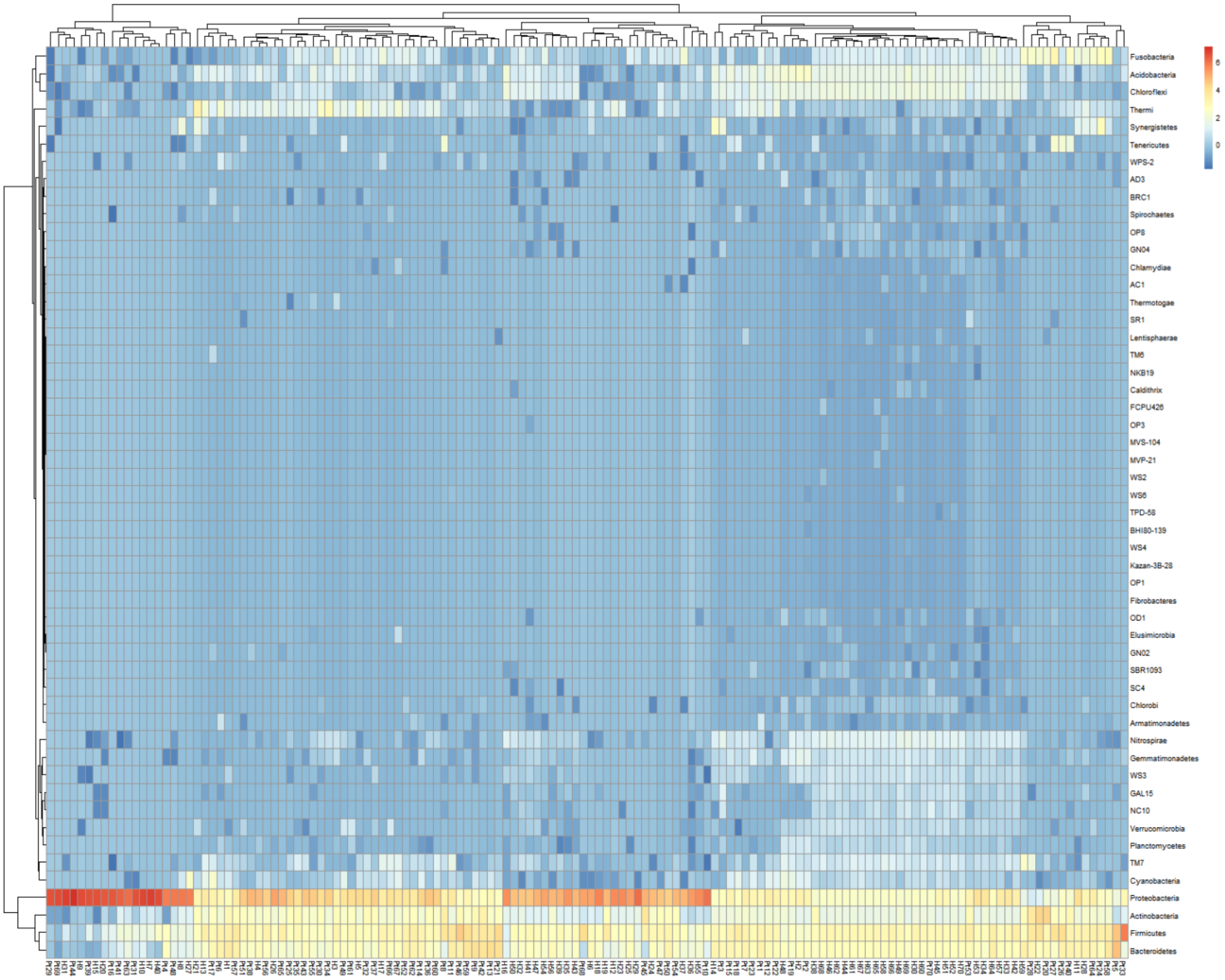

Figure S1: Heatmap at phylum level. Hierarchical clustering was performed, and a heatmap was generated using a Spearman's rank correlation coefficient as a distance measure, as well as a customized script developed in the R statistical package. The heatmap was based on log (2) transformed values of read counts for OTUs in the two cohorts.

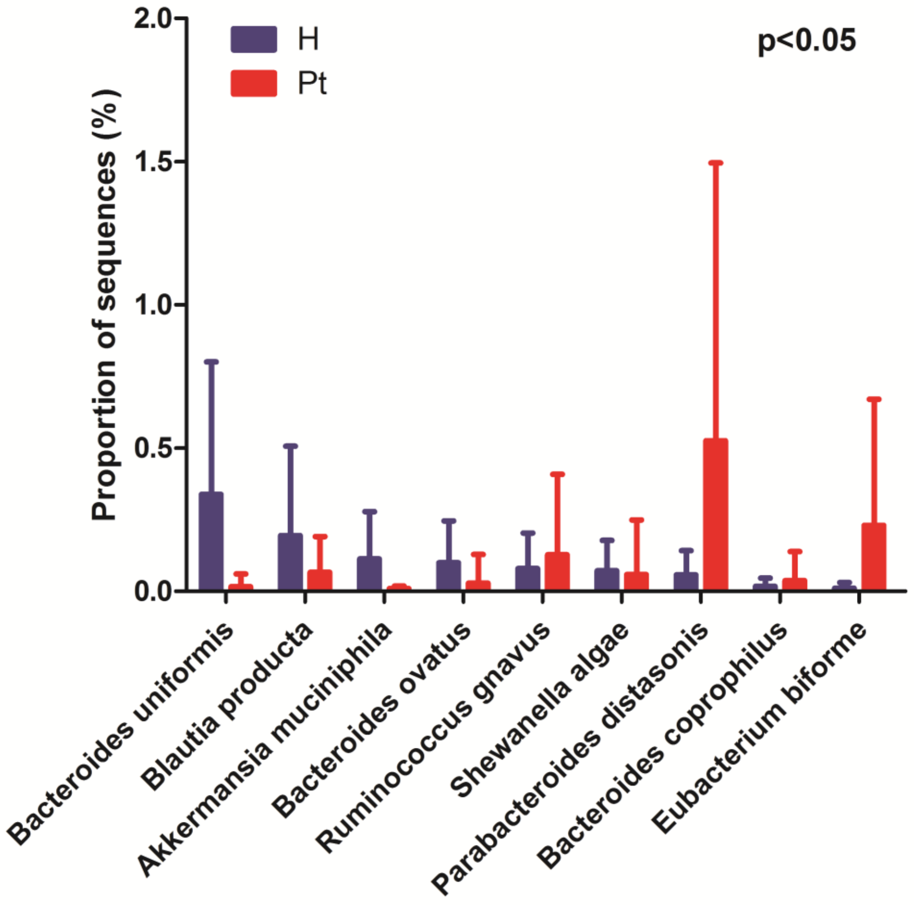

Figure S2: Species-level OTUs different between the two cohorts (Mean ± SD). The STAMP software was used to calculate the bacterial species proportions in the two cohorts. Welch's *t*-test was used to compare abundance at the bacterial genus level for HCs and T2DM patients. The different genera were assigned only to those presenting a minimum variation at a significant level [p (corrected) < 0.05)]. H and Pt represent healthy controls and T2DM patients, respectively.

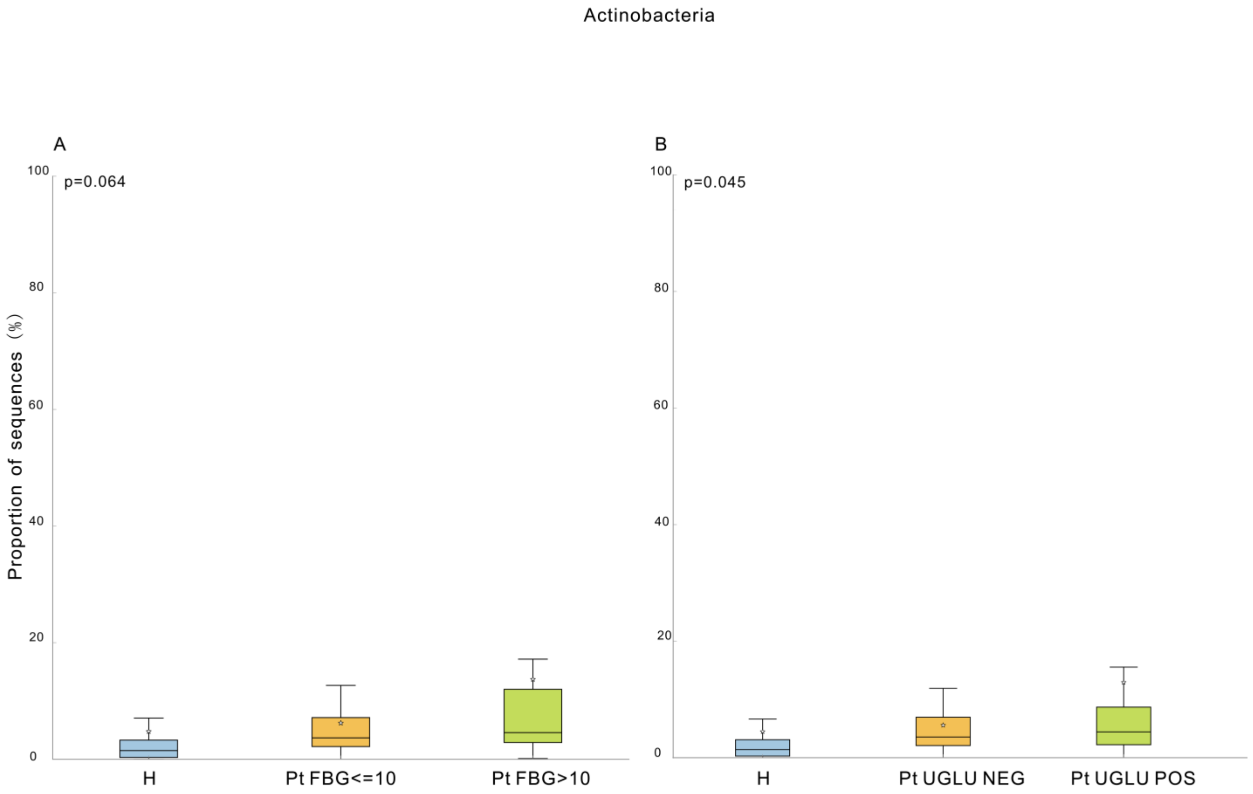

Figure S3: The relative abundance of Actinobacteria associated with FBG and UGLU. (A) Box plot showing the distribution in the proportion of Actinobacteria assigned to samples from H, Pt FBG < = 10 mmol/L, Pt FBG > 10 mmol/L. (B) Samples from H, Pt UGLU NEG, Pt UGLU POS. The median value is shown as a line within the box and the mean value as a star. ANOVA test was applied, and Benjamini-Hochberg FDR was used as a correction approach to control the false discovery rate. P (corrected) < 0.05 was considered significant. H and Pt represent healthy controls and T2DM patients, respectively. UGLU NEG and UGLU POS stand for urine glucose negative and positive in T2DM patients.

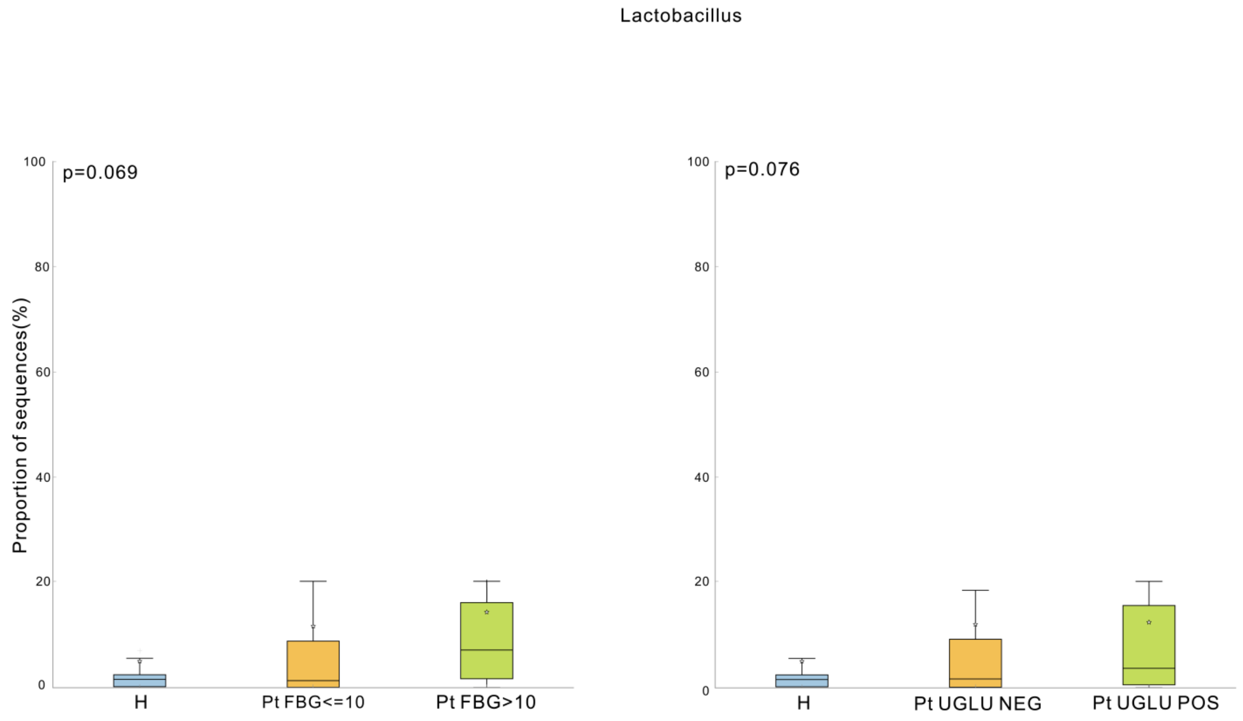

Figure S4: Relative abundance of *Lactobacillus* associated with FBG and UGLU. (A) Box plot showing the distribution in the proportion of *Lactobacillus* assigned to samples from H, Pt FBG < = 10 mmol/L, Pt FBG > 10 mmol/L. (B) Samples from H, Pt UGLU NEG, Pt UGLU POS. The median value is shown as a line within the box, and the mean value as a star. ANOVA test was applied. $P < 0.05$ was considered significant. H and Pt represent healthy controls and T2DM patients, respectively. UGLU NEG and UGLU POS stand for urine glucose negative and positive in T2DM patients.

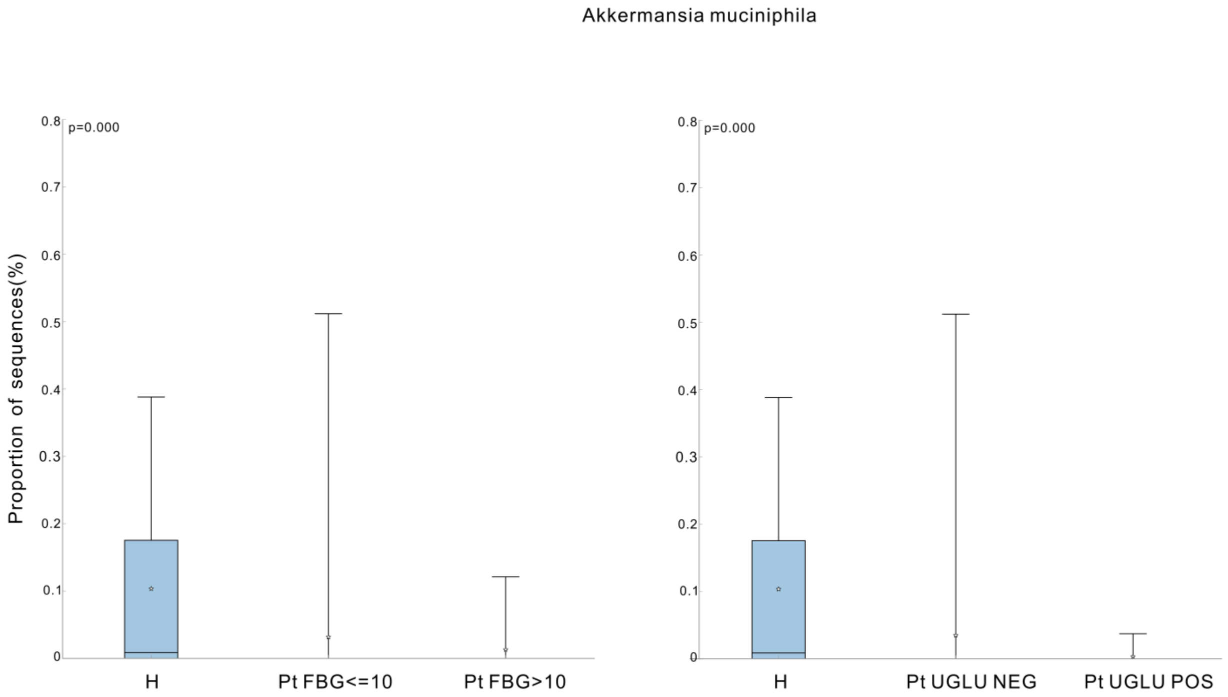

Figure S5: The relative abundance of *Akkermansia muciniphila* associated with FBG and UGLU.

(A) Box plot showing the distribution in the proportion of *Akkermansia muciniphila* assigned to samples from H, Pt FBG < = 10, and Pt FBG > 10. (B) Samples from H, Pt UGLU NEG, and Pt UGLU POS. The median value is shown as a line within the box, and the mean value as a star. ANOVA test was applied, and Benjamini-Hochberg FDR was used as a correction approach to control the false discovery rate. P (corrected) < 0.05 was considered significant. H and Pt represent healthy controls and T2DM patients, respectively. UGLU NEG and UGLU POS stand for urine glucose negative and positive in T2DM patients.

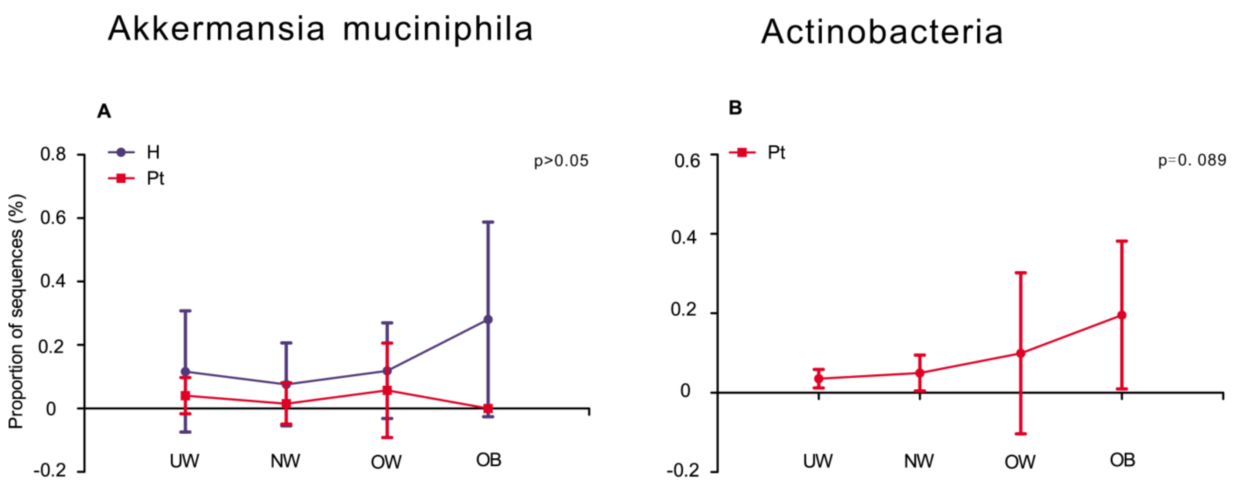

Figure S6: Relative abundance of *Akkermansia muciniphila* and Actinobacteria associated with BMI.

(A) Variation in the abundance of *Akkermansia muciniphila* in different BMI subgroups in HCs and T2DM patients. (B) Variation of the abundance of Actinobacteria in different BMI subgroups in the T2DM cohort. The differences among underweight (UW), normal weight (NW), overweight (OW), and obese (OB) subjects. The WHO-Asian criteria was used to refer to UW (18.5 < BMI), NW (18.5 ≤ BMI < 23), overweight (23 ≤ BMI < 25), and OB (BMI ≥ 25). ANOVA test was applied. H and Pt represent healthy controls and T2DM patients, respectively.

For supplementary Tables see in online.

Table S1: Microbial richness and diversity metrics in the individual urinary microbiota.

[a] The parameters were calculated by QIIME software;

[b] the operational taxonomic units (OTUs) were defined at the 97% similarity level;

[c] H, Pt, and ACE represents healthy controls, T2DM patients, and Abundance-based Coverage Estimator, respectively.

Table S2: Correlation relationship between bacterial diversity, richness and age in two cohorts.

Pearson correlation analysis was performed.

Tables S3: Proportion of bacteria genera in HCs and T2DM cohorts.

The STAMP software was used to calculate the bacterial genus proportions in the two cohorts. If the bacterial genus' relative abundance was less than 1%, it was filtered from the list. Welch's t-test and corrected $p < 0.05$ was applied.
