# Supplementary material for: Dysbiosis of urinary microbiota is positively correlated with Type 2 diabetes mellitus
Source: Oncotarget. 2016 Dec 19;8(3):3798–810. doi: 10.18632/oncotarget.14028 (PMC5354796; doi:10.18632/oncotarget.14028)
Supplement: Supplementary file 2 [file oncotarget-08-3798-s002.docx]

**Table S1.** Microbial richness and diversity metrics in the individual urinary microbiota

| **Sample ID** | **No. of reads ^a^** | **No. of OTUs ^a, b^** | **ACE ^a^** | **Chao1 ^a^** | **Shannon ^a^** | **Simpson ^a^** |
| --- | --- | --- | --- | --- | --- | --- |
| H01 ^c^ | 40419 | 1509 | 1895 | 1792 | 5.96 | 0.93 |
| H02 | 24723 | 4054 | 5108 | 4603 | 10.47 | 1.00 |
| H03 | 26249 | 868 | 1473 | 1353 | 5.50 | 0.94 |
| H04 | 24985 | 1006 | 3497 | 3317 | 2.50 | 0.41 |
| H05 | 55829 | 1516 | 4956 | 4184 | 3.01 | 0.51 |
| H06 | 66009 | 1608 | 5385 | 4590 | 1.83 | 0.33 |
| H07 | 57255 | 1305 | 5060 | 4691 | 1.46 | 0.27 |
| H08 | 58907 | 957 | 3502 | 2896 | 1.16 | 0.22 |
| H09 | 80616 | 1070 | 4241 | 3610 | 1.05 | 0.20 |
| H10 | 41795 | 755 | 1130 | 973 | 2.81 | 0.62 |
| H11 | 73334 | 1798 | 2472 | 2190 | 4.85 | 0.88 |
| H12 | 64004 | 1280 | 4344 | 3917 | 1.47 | 0.28 |
| H13 | 30864 | 1095 | 3372 | 2860 | 4.13 | 0.80 |
| H14 | 84225 | 2381 | 3736 | 3297 | 5.15 | 0.91 |
| H15 | 81111 | 1873 | 7269 | 6122 | 1.56 | 0.28 |
| H16 | 26984 | 983 | 4424 | 4111 | 1.65 | 0.29 |
| H17 | 36080 | 2313 | 6066 | 5397 | 5.97 | 0.85 |
| H18 | 41517 | 1185 | 4396 | 4003 | 1.58 | 0.26 |
| H19 | 58145 | 1525 | 3357 | 2981 | 2.76 | 0.54 |
| H20 | 78248 | 2146 | 3893 | 3341 | 4.05 | 0.82 |
| H21 | 50073 | 2728 | 3820 | 3369 | 6.08 | 0.87 |
| H22 | 78136 | 993 | 936 | 805 | 2.25 | 0.67 |
| H23 | 57103 | 1192 | 4819 | 4394 | 1.42 | 0.25 |
| H24 | 91138 | 1898 | 4341 | 3807 | 3.75 | 0.78 |
| H25 | 72769 | 1410 | 5022 | 4329 | 1.49 | 0.27 |
| H26 | 95608 | 2097 | 4771 | 4101 | 2.88 | 0.65 |
| H27 | 73081 | 1957 | 6854 | 5968 | 2.47 | 0.48 |
| H28 | 117953 | 2241 | 3636 | 3184 | 5.65 | 0.94 |
| H29 | 59684 | 1048 | 4365 | 3975 | 2.16 | 0.47 |
| H30 | 63515 | 6943 | 10883 | 9804 | 10.10 | 0.99 |
| H31 | 54797 | 1337 | 1895 | 1666 | 4.15 | 0.75 |
| H32 | 81283 | 2935 | 8629 | 7836 | 2.33 | 0.35 |
| H33 | 62515 | 4047 | 7020 | 6865 | 6.26 | 0.88 |
| H34 | 91809 | 3932 | 8639 | 8328 | 3.53 | 0.52 |
| H35 | 68313 | 1854 | 2532 | 2241 | 4.94 | 0.90 |
| H36 | 173171 | 3806 | 12355 | 11051 | 1.91 | 0.32 |
| H37 | 127429 | 4265 | 13353 | 11991 | 2.51 | 0.38 |
| H38 | 67419 | 4401 | 5550 | 5688 | 7.31 | 0.91 |
| H39 | 111213 | 3313 | 7838 | 6610 | 3.17 | 0.60 |
| H40 | 89875 | 1505 | 3327 | 2660 | 5.83 | 0.91 |
| H41 | 109268 | 3384 | 7548 | 7501 | 2.40 | 0.36 |
| H42 | 46393 | 3526 | 6285 | 6276 | 6.68 | 0.92 |
| H43 | 80412 | 3429 | 9713 | 8735 | 2.92 | 0.43 |
| H44 | 43010 | 3785 | 5629 | 6065 | 9.18 | 0.99 |
| H45 | 59762 | 4690 | 7393 | 7106 | 8.29 | 0.95 |
| H46 | 44214 | 4221 | 6581 | 6719 | 9.25 | 0.99 |
| H47 | 97586 | 3156 | 6143 | 5318 | 2.65 | 0.43 |
| H48 | 308123 | 2808 | 3143 | 2795 | 10.22 | 1.00 |
| H49 | 46545 | 4019 | 5965 | 6316 | 9.30 | 0.99 |
| H50 | 108776 | 3962 | 11121 | 10623 | 2.54 | 0.38 |
| H51 | 36140 | 3364 | 5638 | 5653 | 8.05 | 0.96 |
| H52 | 27442 | 2880 | 4155 | 4361 | 8.99 | 0.99 |
| H53 | 126610 | 5427 | 8989 | 8263 | 5.32 | 0.84 |
| H54 | 73467 | 3578 | 8801 | 7853 | 5.60 | 0.91 |
| H55 | 83603 | 3147 | 7433 | 6640 | 4.92 | 0.85 |
| H56 | 76949 | 3683 | 8812 | 8039 | 5.84 | 0.92 |
| H57 | 69200 | 4561 | 7876 | 8028 | 6.28 | 0.83 |
| H58 | 42959 | 3617 | 5314 | 5486 | 8.85 | 0.99 |
| H59 | 63815 | 1573 | 2573 | 2417 | 3.12 | 0.68 |
| H60 | 52347 | 4110 | 6008 | 6291 | 9.23 | 0.99 |
| H61 | 46337 | 3922 | 5892 | 6183 | 9.22 | 0.99 |
| H62 | 29624 | 3206 | 4738 | 4915 | 9.17 | 0.99 |
| H63 | 22968 | 2756 | 4019 | 4146 | 9.03 | 0.99 |
| H64 | 42199 | 2330 | 3746 | 3659 | 6.17 | 0.90 |
| H65 | 26006 | 2896 | 4195 | 4462 | 9.10 | 0.99 |
| H66 | 17586 | 2483 | 4084 | 4047 | 9.03 | 0.99 |
| H67 | 17548 | 2474 | 3878 | 3808 | 9.08 | 0.99 |
| H68 | 25974 | 2982 | 4445 | 4580 | 9.09 | 0.99 |
| H69 | 33422 | 4506 | 6605 | 6299 | 10.16 | 1.00 |
| H70 | 24831 | 2849 | 4333 | 4428 | 8.49 | 0.98 |
| Pt01 ^c^ | 59683 | 2413 | 5102 | 4822 | 7.34 | 0.98 |
| Pt02 | 72614 | 9288 | 9875 | 9424 | 11.05 | 1.00 |
| Pt03 | 29481 | 1921 | 2622 | 2457 | 7.25 | 0.96 |
| Pt04 | 80113 | 961 | 1146 | 1042 | 2.72 | 0.64 |
| Pt05 | 68232 | 953 | 1844 | 1622 | 2.97 | 0.73 |
| Pt06 | 69734 | 1906 | 3618 | 3280 | 6.95 | 0.98 |
| Pt07 | 25440 | 1327 | 1727 | 1658 | 7.72 | 0.98 |
| Pt08 | 63676 | 1443 | 1884 | 1725 | 3.57 | 0.72 |
| Pt09 | 42766 | 1624 | 2099 | 1975 | 5.10 | 0.79 |
| Pt10 | 119995 | 1459 | 1869 | 1701 | 1.72 | 0.29 |
| Pt11 | 32854 | 1445 | 1939 | 1830 | 6.61 | 0.93 |
| Pt12 | 76683 | 2584 | 5530 | 5070 | 6.82 | 0.93 |
| Pt13 | 43949 | 1266 | 1322 | 1226 | 4.68 | 0.86 |
| Pt14 | 38894 | 1103 | 1565 | 1455 | 3.71 | 0.59 |
| Pt15 | 48297 | 2697 | 4695 | 4435 | 8.76 | 0.99 |
| Pt16 | 137216 | 1481 | 1610 | 1522 | 2.31 | 0.40 |
| Pt17 | 80581 | 2359 | 4658 | 4325 | 6.57 | 0.94 |
| Pt18 | 144085 | 3848 | 6869 | 6285 | 6.15 | 0.88 |
| Pt19 | 20196 | 1133 | 1294 | 1232 | 7.44 | 0.96 |
| Pt20 | 29971 | 737 | 965 | 859 | 3.20 | 0.71 |
| Pt21 | 72981 | 5613 | 8182 | 7325 | 7.92 | 0.95 |
| Pt22 | 26610 | 1516 | 3143 | 2955 | 7.45 | 0.98 |
| Pt23 | 54167 | 2562 | 4573 | 4276 | 8.39 | 0.99 |
| Pt24 | 40053 | 790 | 1153 | 1095 | 5.40 | 0.95 |
| Pt25 | 110709 | 3706 | 11304 | 10459 | 2.99 | 0.48 |
| Pt26 | 19154 | 537 | 990 | 954 | 3.82 | 0.81 |
| Pt27 | 40765 | 960 | 2405 | 2163 | 3.54 | 0.77 |
| Pt28 | 91113 | 1723 | 1899 | 1723 | 3.80 | 0.83 |
| Pt29 | 111708 | 1265 | 4816 | 4173 | 1.01 | 0.20 |
| Pt30 | 55521 | 2647 | 6513 | 6620 | 4.21 | 0.74 |
| Pt31 | 71343 | 1339 | 5713 | 5072 | 1.22 | 0.22 |
| Pt32 | 47439 | 2072 | 5815 | 5329 | 4.12 | 0.72 |
| Pt33 | 30884 | 872 | 2325 | 1911 | 1.82 | 0.42 |
| Pt34 | 33075 | 1333 | 3470 | 3334 | 2.70 | 0.47 |
| Pt35 | 49945 | 1644 | 3950 | 3711 | 3.35 | 0.60 |
| Pt36 | 51632 | 1412 | 4258 | 3956 | 2.51 | 0.44 |
| Pt37 | 40687 | 1464 | 3152 | 2976 | 2.92 | 0.49 |
| Pt38 | 37481 | 1391 | 4536 | 4873 | 2.54 | 0.43 |
| Pt39 | 102341 | 1548 | 6714 | 5452 | 1.21 | 0.23 |
| Pt40 | 29100 | 756 | 1673 | 1423 | 4.50 | 0.90 |
| Pt41 | 141907 | 2789 | 8992 | 7560 | 1.58 | 0.28 |
| Pt42 | 60660 | 1115 | 1621 | 1495 | 4.54 | 0.88 |
| Pt43 | 60756 | 1550 | 4885 | 4388 | 2.53 | 0.44 |
| Pt44 | 97640 | 1167 | 4447 | 3919 | 1.10 | 0.23 |
| Pt45 | 52491 | 2149 | 4262 | 3719 | 3.81 | 0.73 |
| Pt46 | 58759 | 712 | 816 | 754 | 2.93 | 0.70 |
| Pt47 | 38512 | 999 | 4468 | 4335 | 2.47 | 0.46 |
| Pt48 | 90526 | 1263 | 4937 | 4302 | 1.18 | 0.23 |
| Pt49 | 15357 | 789 | 2506 | 2328 | 3.65 | 0.61 |
| Pt50 | 53029 | 1469 | 5086 | 4636 | 2.76 | 0.49 |
| Pt51 | 53887 | 1983 | 6220 | 6238 | 3.02 | 0.52 |
| Pt52 | 24950 | 1217 | 3017 | 2921 | 5.05 | 0.79 |
| Pt53 | 61825 | 3348 | 6558 | 5856 | 6.86 | 0.96 |
| Pt54 | 36744 | 864 | 2708 | 2741 | 1.87 | 0.34 |
| Pt55 | 27821 | 1001 | 3177 | 2873 | 2.29 | 0.40 |
| Pt56 | 13419 | 493 | 1416 | 1535 | 2.70 | 0.49 |
| Pt57 | 17696 | 656 | 1436 | 1296 | 5.07 | 0.93 |
| Pt58 | 54188 | 924 | 1479 | 1420 | 4.17 | 0.84 |
| Pt59 | 35125 | 621 | 1102 | 958 | 2.82 | 0.69 |
| Pt60 | 26446 | 939 | 2998 | 2452 | 2.95 | 0.51 |
| Pt61 | 28313 | 1145 | 3878 | 3453 | 3.59 | 0.58 |
| Pt62 | 47096 | 1398 | 3792 | 3389 | 4.62 | 0.85 |
| Pt63 | 146528 | 2937 | 8766 | 7570 | 1.89 | 0.33 |
| Pt64 | 25175 | 841 | 2006 | 2063 | 4.95 | 0.89 |
| Pt65 | 23647 | 1039 | 3990 | 3685 | 2.62 | 0.44 |
| Pt66 | 32635 | 1371 | 4505 | 4001 | 4.02 | 0.64 |
| Pt67 | 42859 | 1720 | 5079 | 4841 | 4.93 | 0.81 |
| Pt68 | 86927 | 2169 | 8100 | 6918 | 2.25 | 0.46 |
| Pt69 | 111251 | 1424 | 5764 | 5050 | 1.14 | 0.23 |
| Pt70 | 14182 | 2402 | 3993 | 3816 | 9.26 | 0.99 |

^a^ The parameters were calculated by QIIME software;

^b^ the operational taxonomic units (OTUs) were defined at the 97% similarity level;

^c^ H, Pt, and ACE represents healthy controls, T2DM patients, and Abundance-based Coverage Estimator, respectively.
