# Supplementary material for: Dysbiosis of urinary microbiota is positively correlated with Type 2 diabetes mellitus
Source: Oncotarget. 2016 Dec 19;8(3):3798–810. doi: 10.18632/oncotarget.14028 (PMC5354796; doi:10.18632/oncotarget.14028)
Supplement: Supplementary file 3 [file oncotarget-08-3798-s003.doc]

**Table S2.** Correlation relationship between bacterial diversity, richness and age in two cohorts

| **Parameter** | **HCs** | |  | **T2DM** | |
| --- | --- | --- | --- | --- | --- |
|  | **r** | **p-value** |  | **r** | **p-value** |
| OTUs | -0.20 | 0.106 |  | -0.15 | 0.207 |
| Chao1 | -0.19 | 0.118 |  | -0.18 | 0.132 |
| Shannon | 0.00 | 0.989 |  | -0.03 | 0.835 |
| Simpson | -0.10 | 0.932 |  | -0.02 | 0.859 |

Pearson correlation analysis was performed.
