# Supplementary material for: Dysbiosis of urinary microbiota is positively correlated with Type 2 diabetes mellitus
Source: Oncotarget. 2016 Dec 19;8(3):3798–810. doi: 10.18632/oncotarget.14028 (PMC5354796; doi:10.18632/oncotarget.14028)
Supplement: Supplementary file 4 [file oncotarget-08-3798-s004.docx]

**Table S3**. Proportion of bacteria genera in HCs and T2DM cohorts

| **Taxon** | **HC** | **T2DM** |
| --- | --- | --- |
| Prevotella | 12.67 | 18.76 |
| Lactobacillus | 5.04 | 12.15 |
| Shuttleworthia | 1.53 | 6.42 |
| Streptococcus | 1.24 | 6.07 |
| Acinetobacter | 1.20 | 3.74 |
| Bacteroides | 6.02 | 3.43 |
| Halomonas | 1.31 | 2.79 |
| Blautia | 7.24 | 2.17 |
| Faecalibacterium | 2.90 | 1.45 |
| Corynebacterium | 0.68 | 1.30 |
| Klebsiella | 7.07 | 0.93 |
| Pseudomonas | 6.40 | 0.86 |
| Coprococcus | 1.64 | 0.85 |
| Phascolarctobacterium | 1.98 | 0.80 |
| Dorea | 1.73 | 0.79 |
| Shewanella | 0.29 | 0.69 |
| Megamonas | 1.44 | 0.52 |
| Oscillospira | 0.72 | 0.36 |
| Actinomyces | 0.10 | 0.25 |
| Finegoldia | 0.09 | 0.23 |
| Lachnospira | 0.58 | 0.22 |
| Staphylococcus | 0.07 | 0.22 |
| Microbacterium | 0.05 | 0.22 |
| Sutterella | 0.36 | 0.19 |
| Gemella | 0.03 | 0.16 |
| Akkermansia | 0.26 | 0.11 |
| Anaerostipes | 0.20 | 0.09 |
| Bilophila | 0.12 | 0.05 |
| Brevibacterium | 0.01 | 0.04 |
| Odoribacter | 0.10 | 0.03 |
| Paraprevotella | 0.07 | 0.02 |
| Butyricimonas | 0.06 | 0.02 |
| Lachnobacterium | 0.26 | 0.02 |

The STAMP software was used to calculate the bacterial genus proportions in the two cohorts. If the bacterial genus’ relative abundance was less than 1%, it was filtered from the list. Welch’s t-test and corrected p < 0.05 was applied.
